# Complete Genome Sequences of Two Japanese Eel Endothelial Cell-Infecting Virus Strains Isolated in Japan

**DOI:** 10.1128/genomeA.01236-15

**Published:** 2015-11-12

**Authors:** Yuki Naoi, Sachiko Okazaki, Yukie Katayama, Tsutomu Omatsu, Shin-ichi Ono, Tetsuya Mizutani

**Affiliations:** a Research and Education Center for Prevention of Global Infectious Diseases of Animals, Tokyo University of Agriculture and Technology, Fuchu, Tokyo, Japan; b The United Graduate School of Veterinary Sciences, Gifu University, Gifu, Japan; c School of Marine Science and Technology, Tokai University, Shimizu-ku, Shizuoka, Japan

## Abstract

Japanese eel endothelial cell-infecting virus (JEECV) causes viral endothelial cell necrosis of eel (VECNE), resulting in severe economic losses in eel aquaculture in Japan. Here, we report the complete genome sequences of two new JEECV strains isolated from farmed Japanese eels.

## GENOME ANNOUNCEMENT

Japanese eel endothelial cell-infecting virus (JEECV) is one of the most important viruses in the Japanese eel, *Anguilla japonica* (Temminck and Schlegel, 1847). JEECV causes viral endothelial cell necrosis of eel (VECNE) (1, 2), which is a lethal disease involving hemorrhage and congestion in the central venous sinus of the gills of farmed eels (3).

JEECV is a double-stranded circular DNA virus. It is thought to be a chimeric virus between a polyomavirus and a virus of an unknown family, because the JEECV genome without the polyomavirus large T-like protein gene (LTLG) does not exhibit homology with any virus (2). We also detected JEECVs with a single amino acid change at the LTLG in Japanese eels with no symptoms and that are living in natural habitats (4). However, to date, JEECV genome analyses have only used 70th-passage viruses (2). Here, we report the complete genome sequence of two wild-type JEECVs obtained from farmed eels with VECNE and compare it with that of a previously reported reference strain.

JEECVs were isolated from farmed eels with VECNE in the Shizuoka and Tokushima prefectures. The virus strains were passaged <10 times in Japanese eel endothelial cells (1). Viral DNA was extracted from the supernatants and randomly amplified using the GenomiPhi version 2 kit (GE Healthcare). The library for deep sequencing was prepared using the Nextera XT DNA sample prep kit (Illumina), and sequencing was conducted on the MiSeq (Illumina) benchtop sequencer. Bioinformatic analysis was conducted using the CLC Genomics Workbench 6.5.1 (CLC bio). Following *de novo* assembly, the contigs were mapped to the complete JEECV genome (GenBank accession no. AB543063). In both strains (one from each prefecture), three contigs were mapped, measuring 9,309, 3,475, and 1,363 nucleotides (nt) in the Shizuoka strain, and 9,280, 3,560, and 1,981 nt in the Tokushima strain. Three unidentified regions were amplified using specific primers. One of the three amplicons, a region containing a 105-nt-repeat-sequence, was determined by Sanger sequencing on 3130xl DNA analyzer (Applied Biosystems). Finally, all obtained sequences were aligned and manually reassembled.

The genomes of the Shizuoka and Tokushima strains comprise 14,986 bp and 15,080 bp, respectively. Complete genome alignment revealed that the Shizuoka strain shares 99.33% nucleotide identity with the Tokushima strain, although the two viruses were isolated from geographically distinct places. Each strain had two long (≥10 nt) deletions: one was a 39-nt deletion within the 2756 to 2794 nt region of the reference strain, and the other deletion was located in the 105-nt-repeat region at 487 to 801 nt. While the reference strain has three repeats of 315 bp, the Tokushima strain had a 10-nt deletion in a repeat, and the Shizuoka strain had a 105-nt deletion. LTLGs were completely conserved between the reference strain and the two strains in this report. These data will be helpful in advancing investigations into JEECV pathogenicity.

### Nucleotide sequence accession numbers.

The complete genome sequences of the JEECV Shizuoka and Tokushima strains have been deposited DDBJ/ENA/GenBank under the accession numbers LC081214 and LC081215, respectively.
